# Treatment of Localized and Locally Advanced, High-Risk Prostate Cancer: A Report From the First Prostate Cancer Consensus Conference for Developing Countries

**DOI:** 10.1200/GO.20.00421

**Published:** 2021-04-15

**Authors:** Raja Khauli, Robson Ferrigno, Gustavo Guimarães, Muhammad Bulbulan, Pedro Luiz Serrano Uson Junior, Bernardo Salvajoli, Daniel Moore Freitas Palhares, Douglas Racy, Erlon Gil, Fernando Freire de Arruda, Gustavo Caserta Lemos, Gustavo Franco Carvalhal, Icaro Thiago de Carvalho, Igor Austin Fernandes Martins, Ivan Frederico Pinto Gimpel, João Victor Salvajoli, José Luis Chambo, José Pontes Jr, Leopoldo Alves Ribeiro Filho, Lucas Nogueira, Marcelo Roberto Pereira Freitas, Marcelo Wroclawski, Marco Antonio Arap, Marcus Vinicius Sadi, Rafael Coelho, Rafael Gadia, Rodrigo Antonio Ledezma Roja, Rodrigo de Moraes Hanriot, Ronaldo Baroni, Stenio Zequi, William Carlos Nahas, Wladimir Alfer Jr, Fernando Cotait Maluf

**Affiliations:** ^1^ Naef K. Basile Cancer Institute, Beirut, Lebanon; ^2^ Hospital BP, São Paulo, Brazil; ^3^ American University of Beirut, Beirut, Lebanon; ^4^ Hospital Israelita Albert Einstein, São Paulo, Brazil; ^5^ Instituto do Câncer de São Paulo, São Paulo, Brazil; ^6^ Hospital do Coração, São Paulo, Brazil; ^7^ Hospital Sírio Libanês, São Paulo, Brazil; ^8^ Hospital Moinho de Ventos, Porto Alegre, Brazil; ^9^ Clínica Santa María, Providencia, Chile; ^10^ Universidade Federal de Minas Gerais, Belo Horizonte, Brazil; ^11^ Centro de Pesquisas Oncológicas de Santa Catarina, Santa Catarina, Brazil; ^12^ Universidad de Chile, Santiago, Chile; ^13^ Hospital Alemão Oswaldo Cruz, São Paulo, Brazil; ^14^ Hospital do Câncer AC Camargo, São Paulo, Brazil; ^15^ Latin American Oncology Group (LACOG), Porto Alegre, Brazil

## Abstract

**PURPOSE:**

To generate and present survey results on important issues relevant to treatment and follow-up of localized and locally advanced, high-risk prostate cancer (PCa) focusing on developing countries.

**METHODS:**

A panel of 99 PCa experts developed more than 300 survey questions of which 67 questions concern the main areas of interest of this article: treatment and follow-up of localized and locally advanced, high-risk PCa in developing countries. A larger panel of 99 international multidisciplinary cancer experts voted on these questions to create the recommendations for treatment and follow-up of localized and locally advanced, high-risk PCa in areas of limited resources discussed in this article.

**RESULTS:**

The panel voted publicly but anonymously on the predefined questions. Each question was deemed consensus if 75% or more of the full panel had selected a particular answer. These answers are based on panelist opinion and not on a literature review or meta-analysis. For questions that refer to an area of limited resources, the recommendations considered cost-effectiveness as well as the possible therapies with easier and greater access. Each question had five to seven relevant answers including two nonanswers. Results were tabulated in real time.

**CONCLUSION:**

The voting results and recommendations presented in this article can guide physicians managing localized and locally advanced, high-risk PCa in areas of limited resources. Individual clinical decision making should be supported by available data; however, as guidelines for treatment of localized and locally advanced, high-risk PCa in developing countries have not been defined, this article will serve as a point of reference when confronted with this disease.

## INTRODUCTION

High-risk prostate cancer (PCa) accounts for 15% of cancer diagnoses.^1^ This percentage may be higher in developing countries, especially where multidisciplinary care is limited to few academic medical centers and there are major access barriers from screening to confirmation diagnosis. This article will summarize the recommendations of a large panel of physicians from developing countries, specializing in PCa, regarding the treatment of patients presenting with high-risk PCa both with and without contemplating the restrictions of limited resources in the decision-making process, with the objective of providing guidance in clinical practice and policy development and modification. The complete methodology of the Prostate Cancer Consensus Conference for Developing Countries including the elaboration process of the questionnaires to guide the panelists, the design of voting sessions, and consensus criteria were presented for the editorial and are valid for all the manuscripts (Data Supplement).

**CONTEXT**

**Key Objective**
Generate a consensus on critical issues relevant to treatment of localized and locally advanced, high-risk prostate cancer (PCa) focusing on developing countries.
**Knowledge Generated**
Definitive treatment for high-risk PCa is achieved either surgically or with radiotherapy and androgen deprivation therapy. Androgen deprivation therapy alone is inadequate. Several factors such as life expectancy, tumor stage, prostate-specific antigen, lymph node involvement, and regional metastasis may affect the decision-making process between surgery and radiation. In this article, we developed discussions on the multiple questions voted on at the first Prostate Cancer Consensus Conference for Developing Countries for high-risk PCa.
**Relevance**
The voting results presented in this article can be used to support the treatment of localized and locally advanced, high-risk PCa in areas of limited resources lacking specific guidelines.

## TREATMENT: LOCALIZED AND LOCALLY ADVANCED, HIGH-RISK PROSTATE CANCER

For patients with life expectancy of > 10-15 years with the diagnosis of localized high-risk PCa, with Gleason score 8-10 and/or prostate-specific antigen (PSA) > 20 ng/mL, most panelists (61.15%) recommended radical prostatectomy plus lymph node dissection for treatment, whereas a little more than one-quarter (27.50%) selected the combination of hormonal therapy and external beam radiation therapy (EBRT). For the same patient in an area of limited resources, panelists reached consensus (74.84%) in recommending radical prostatectomy plus lymph node dissection (where Robot Platform is not available), whereas less than one-quarter (23.46%) recommended the combination of hormonal therapy and EBRT (intensity-modulated radiation therapy [IMRT] not available).

For patients with life expectancy of > 10-15 years with the diagnosis of clinical T3/T4 and/or clinical N+, high-risk PCa, the panelists reached consensus in recommending the combination of hormonal therapy and EBRT (IMRT preferred) with or without brachytherapy (76.54%) as well as in recommending the combination of hormonal therapy and EBRT (IMRT not available) with or without brachytherapy (78.75%) for treatment in an area of limited resources.

For patients with life expectancy of < 10-15 years with the diagnosis of localized high-risk PCa, with Gleason score 8-10 and/or PSA > 20 ng/mL, the panelists almost reached consensus (71.76%) in recommending the combination of hormonal therapy and EBRT (IMRT preferred) as treatment, with some (17.65%) choosing the combination of hormonal therapy, EBRT (IMRT preferred), and brachytherapy. For the same patient in an area of limited resources, the panel reached consensus (77.38%) in recommending the combination of hormonal therapy and EBRT (IMRT not available).

The treatment for patients with life expectancy of < 10-15 years with the diagnosis of high-risk PCa with clinical T3/T4 and/or clinical N+, the panel reached consensus (79.76%) in recommending the combination of hormonal therapy and EBRT (IMRT preferred), including in an area of limited resources (85.54%) (IMRT not available).

The panel reached consensus (79.01%) in recommending the combination of hormonal therapy and conformal EBRT for patients with the diagnosis of high-risk PCa with clinical T3/T4 and/or clinical N+ in institutions where there is no availability of IMRT technique.

In institutions where there is no availability of IMRT technique and conformal EBRT, the panel also reached consensus (82.35%) in recommending radical prostatectomy plus lymph node dissection for patients with the diagnosis of high-risk PCa with Gleason score 8-10 and/or PSA > 20 ng/mL and disease confined to the prostate.

In institutions where there is only conventional radiation therapy (RT) technique, for patients with high-risk, organ-confined PCa, the panel reached consensus (78.31%) in recommending external RT. In institutions where there is only cobalt RT technique, the panel reached strong consensus (89.95%) in recommending that patients with high-risk, disease-confined PCa cannot be treated with external RT.

In the case where some form of radiation is an option for treatment of a high-risk patient with PCa with life expectancy of > 10-15 years, the panel agreed, and in most cases reached consensus, on a recommendation for the association of hormonal treatment under varying circumstances. In the case where radical prostatectomy is an option for treatment for the same patient, the panel recommended extended lymph node dissection, in all circumstances (Tables 1 and 2).

**TABLE 1:**
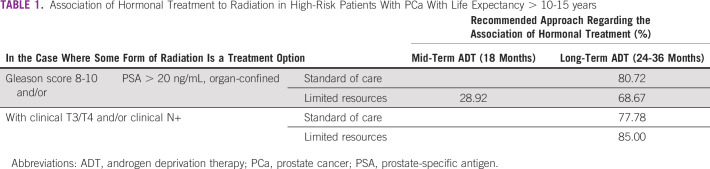
Association of Hormonal Treatment to Radiation in High-Risk Patients With PCa With Life Expectancy > 10-15 years

**TABLE 2:**
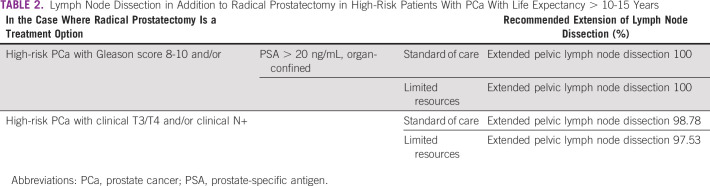
Lymph Node Dissection in Addition to Radical Prostatectomy in High-Risk Patients With PCa With Life Expectancy > 10-15 Years

In the case where radical prostatectomy is an option for treatment of a patient with life expectancy of > 10-15 with a diagnosis of high-risk PCa, the panel reached consensus in recommending a surgical approach under varying circumstances. The panel reached strong consensus in recommending an open radical surgery in areas of limited resources (Table 3).

**TABLE 3:**
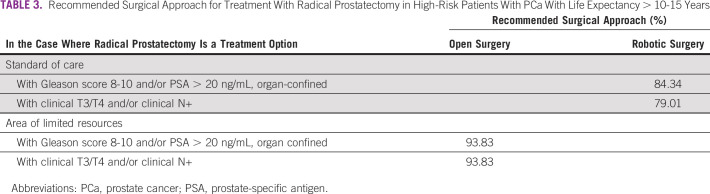
Recommended Surgical Approach for Treatment With Radical Prostatectomy in High-Risk Patients With PCa With Life Expectancy > 10-15 Years

In the case where exclusive hormonal therapy is an option for treatment of high-risk PCa, the panel had differing recommendations for their preferred treatment under varying circumstances and reached consensus in only one instance, the application of orchiectomy alone as a means of androgen deprivation therapy (ADT) for patients with clinical T3/T4 and/or clinical N+ in areas of limited resources (Table 4).

**TABLE 4:**
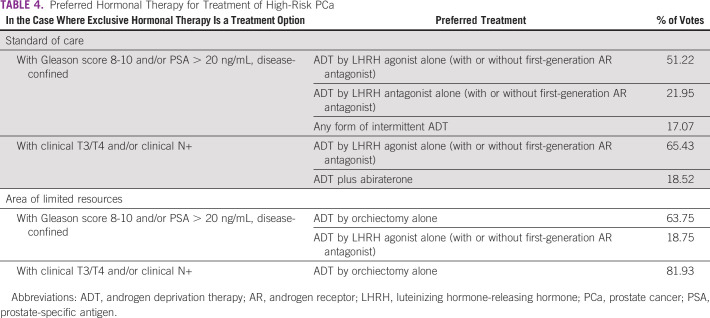
Preferred Hormonal Therapy for Treatment of High-Risk PCa

## RADIOTHERAPY

Over the past few decades, radiation techniques have improved, allowing better coverage of tumor volumes with improved sparing of adjacent normal structures.^2,3^ EBRT is the most used type of radiation treatment, in particular IMRT. The enhanced conformity of IMRT allows for dose escalation to the prostate while reducing the dose to the bladder and rectum, and trials have demonstrated reduced toxicity with IMRT; however, reimbursements for IMRT were higher, leading to increased costs in the overall care for PCa.^4^ When comparing different radiotherapy strategies, including three-dimensional conformal radiation therapy (3DCRT) with IMRT, IMRT was found to be cost effective in one Australian study, demonstrating an approximate 1.1 million dollars in savings per 1,000 patients.^5^

## ANDROGEN DEPRIVATION THERAPY

RT combined with ADT was the preferred option for high-risk patients with clinical T3/T4 and/or clinical N+ if IMRT or conformal EBRT is available. Despite IMRT being preferred over conformal RT because of reduced late toxicity, the efficacy of both techniques is comparable; therefore, the latter is a reasonable option in the treatment of localized and locally advanced disease, particularly in areas of limited resources where IMRT is not available. A study evaluating two prospective cohorts of men treated for localized PCa investigated the hypothesis of reductions in toxicity and showed that the 5-year cumulative incidence of grade ≥ 2 GI toxicity was 24.9% for IGRT and IMRT and 37.6% with 3DCRT (*P* = .005), with significant reductions in proctitis (*P* = .047) and increased stool frequency (*P* < .001). On the other hand, genitourinary grade ≥ 2 toxicity levels at 5 years were comparable.^6^

The panel favored radical prostatectomy over radiation when neither IMRT nor conformal radiation was available. Cobalt RT in this consensus was defined as using the external beam from a ^60^Cobalt unit, with parallel opposed, three-field or four-field box technique, with anatomic bony landmarks on plain radiographs being used to define shape and location of the prostate. This technique presents target inaccuracies, higher volumes of irradiation to normal tissue, and lower energy used (average beam energy of 1.25 MeV) when compared with modern linear accelerators. Consequently, the panel considered this treatment to have inferior efficacy because of the lower doses toward the tumor and pelvic drainage as well more toxicity.^7^

ADT remains a cornerstone in the treatment of high-risk localized and advanced PCa. In localized and locally advanced disease, ADT is typically combined with RT. The duration of ADT is usually 2- 3 years, based on clinical trials that established that long-term ADT (28-36 months) is more effective than short-term ADT (4-6 months). On the other hand, ADT alone is inferior to ADT combined with radiation in high-risk and/or locally advanced PCa.^8^

ADT alone should be considered in patients with a contraindication to RT or in areas of limited resources with no access to RT. Bilateral orchiectomy is a surgical option for metastatic disease and for selected elderly patients with locally advanced disease.^9^ It is well known that orchiectomy is less expensive than any other ADT therapy. It is estimated that the cost of luteinizing hormone-releasing hormone (LHRH) agonist treatment for androgen suppression is 10-13 times and that for combined androgen blockade is 17-20 times higher than the cost of bilateral orchiectomy.^10^

## RADICAL PROSTATECTOMY

Radical prostatectomy is a second treatment option for patients with high-risk PCa, as noted in the NCCN, American Urological Association, and European Association of Urology guidelines.^11,12^ In addition, in this setting, pelvic lymph node dissection is considered the surgical standard for PCa staging. There are no randomized clinical trials completed comparing surgery versus radiation. Therefore, there is no consensus regarding what the best approach is focusing on efficacy, toxicity, and quality of life. A retrospective German analysis including 910 men with localized disease treated with surgery, 292 with radiation, and 124 with active surveillance concluded that surgery was associated with more life-years gained. However, because of the high inpatient costs of the initial surgery, radical prostatectomy had €11,000 in Euros higher total per capita costs than radiation or active surveillance.^13^ The length of RT compared with surgery may also present an issue in patients with limited transportation and/or access to the health centers.

## ADJUVANT TREATMENT AFTER RADICAL PROSTATECTOMY: LOCALIZED AND LOCALLY ADVANCED, HIGH-RISK PROSTATE CANCER

When considering patients with life expectancy of < 10-15 years or > 10-15 years with adverse factors postprostatectomy (positive margins and/or extracapsular disease and/or seminal vesicle involvement) but no pathologic lymph node involvement (pN0) and undetectable postoperative PSA, the panel differed in their recommendations for adjuvant radiation and hormonal therapy, including in areas of limited resources. Consensus was not reached on any recommendation for these patients (Table 5).

**TABLE 5:**
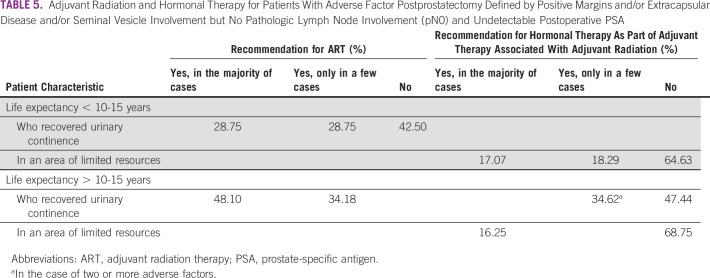
Adjuvant Radiation and Hormonal Therapy for Patients With Adverse Factor Postprostatectomy Defined by Positive Margins and/or Extracapsular Disease and/or Seminal Vesicle Involvement but No Pathologic Lymph Node Involvement (pN0) and Undetectable Postoperative PSA

In line with recent recommendations on the basis of randomized controlled trials, when considering hormonal therapy as an option as part of adjuvant therapy in association with RT because of positive margins and/or extracapsular disease and/or seminal vesicle involvement, although not reaching consensus, most of the panel (72.29%) preferred ADT by LHRH agonist alone (with or without first-generation androgen receptor [AR] antagonist), whereas less than one-fourth (21.69%) recommended no ADT. If adjuvant hormonal therapy alone is recommended for a patient with positive margins and/or extracapsular disease and/or seminal vesicle involvement, slightly more than half (51.76%) of the panel voted to abstain from the answer. Some of the panel members (21.18%) selected long-term ADT (24-36 months) and others (18.82%) chose short-term ADT (6 months).

For the same patient, in an area of limited resources, the panel was almost equally divided in their preference, with one-third (34.18%) recommending ADT by LHRH agonist alone (with or without first-generation AR antagonist), another third (34.18%) indicating no ADT would be recommended, and just less than one-quarter (22.78%) abstaining. If the panelists recommend adjuvant hormonal therapy alone for this patient, as to the preferred duration of hormonal therapy, most panelists (68.67%) abstained, whereas few selected short-term ADT (6 months) and long-term ADT (24-36 months) in combination with radiation.

In institutions where only conventional conformal EBRT is available (no IMRT), the panelists were divided in recommending adjuvant radiation therapy (ART) in men with life expectancy of > 10-15 years who recovered urinary continence, with adverse factors postprostatectomy (positive margins and/or extracapsular disease and/or seminal vesicle involvement but no pathologic lymph node involvement [pN0]) and undetectable postoperative PSA. More than half (48.10%) recommended ART in the majority of the cases. For the same patient, in institutions where only cobalt RT is available, the panel reached consensus (93.83%) in not recommending ART.

For men with life expectancy of > 10-15 years who recovered urinary continence with pathologic lymph node involvement (pN+) and undetectable postoperative PSA, the majority of the panel (54.55%) recommended ART in the majority of cases, whereas almost one-third (31.17%) recommended it only in a few cases.

For men with pathologic lymph node involvement (pN+) and undetectable postoperative PSA, most of the panelists (68.35%) recommended hormonal therapy as part of adjuvant therapy associated with adjuvant radiation in the majority of cases, some (16.48%) did not recommend it, and others (15.19%) recommended it only in a few cases. This poses a trend toward a consensus in that the majority of the panelists (84.83%) recommended hormonal therapy for either all or some cases.

For men with pathologic lymph node involvement (pN+) and undetectable postoperative PSA, most panelists (50.62%) do not recommend isolated hormonal therapy as adjuvant therapy, whereas almost one-third (31.10%) recommend it only in a few cases.

When hormonal therapy is an option as part of adjuvant therapy in association with RT because of pathologic lymph node involvement (pN+), the panelists reached consensus (90.12%) in preferring ADT by LHRH agonist alone (with or without first-generation AR antagonist).

If the panelists recommended adjuvant hormonal therapy because of pathologic lymph node involvement (pN+), most (70.51%) selected long-term ADT, of which 55.13% would give it for 24-36 months and 15.38% recommended ADT indefinitely; 17.95% abstained.

In areas of limited resources, the panelists' opinions differed for recommendations of ART, hormonal therapy as part of adjuvant therapy associated with adjuvant radiation, and isolated hormonal therapy as adjuvant therapy for men under varying circumstances, as noted in Table 6.

**TABLE 6:**
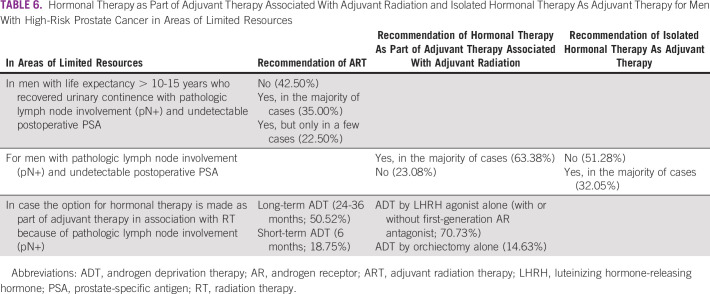
Hormonal Therapy as Part of Adjuvant Therapy Associated With Adjuvant Radiation and Isolated Hormonal Therapy As Adjuvant Therapy for Men With High-Risk Prostate Cancer in Areas of Limited Resources

In institutions where there is only conventional conformal EBRT technique (no IMRT), for men with life expectancy of > 10-15 years who recovered urinary continence, with pathologic lymph node involvement (pN+) and undetectable postoperative PSA, half the panel (50.52%) recommend ART in the majority of cases, whereas one-quarter (26.25%) recommend it in a few cases and one-fifth (20.21%) do not recommend it. For the same patient, in institutions where there is only cobalt RT technique, the panel reached consensus and 95% of the voters would not recommend ART.

In this situation, slightly more than half of the panelists (54.32%) recommended hormonal therapy as part of adjuvant therapy associated with radiation, either conformal or cobalt (no IMRT), for men with pathologic lymph node involvement (pN+) and undetectable postoperative PSA in the majority of cases, whereas almost one-quarter (23.26%) did not.

The panelists were divided when asked about the extension of the RT field in case of ART in men with high-risk PCa (positive margins and/or extracapsular extension and/or seminal vesicle involvement) and pN0 postprostatectomy. Almost half of the panel (45.12%) recommended prostatic bed only and the same percentage (45.12%) recommended prostatic bed plus whole pelvis.

In institutions where there is only conventional conformal EBRT available (no IMRT) and ART is recommended for men with high-risk PCa (positive margins and/or extracapsular extension and/or seminal vesicle involvement) and pN0 postprostatectomy, most panelists (62.96%) recommended to irradiate the prostatic bed only. For the same patient and circumstance, in institutions where there is only cobalt RT technique, the panelists reached consensus (93.83%) in not recommending RT. This recommendation contrasts with recent literature recommending whole-pelvis radiation in addition to prostatic fossa radiation.

When treating with ART in men with pathologic lymph node involvement (pN+) and undetectable postoperative PSA, the panel reached consensus (89.02%) in recommending prostatic bed and whole pelvis as the RT volumes. In institutions where there is only conventional conformal EBRT technique (no IMRT) and ART is recommended in men with pathologic lymph node involvement (pN+) and undetectable postoperative PSA, the panel reached consensus (85.71%) in recommending RT to the prostatic bed plus whole pelvis. For the same patient and circumstance, in institutions where there is only cobalt RT technique, the panel reached almost complete consensus (98.73%) in not recommending RT in this scenario.

For patients with high-risk PCa, most panelists (60%) recommended external iliac, internal iliac, obturator, and common iliac as the pelvic lymph node dissection template, although some (22.35%) recommended external iliac, internal iliac, obturator, common iliac, and presacral. For the same patient in an area of limited resources, the panel made a similar recommendation with most panelists (62.03%) recommending external iliac, internal iliac, obturator, and common iliac.

ART for patients with higher risk of relapse after radical prostatectomy has been studied in several randomized trials with questionable results regarding an overall survival benefit of this strategy.^14-16^ One of the most important questions in this space refers to the optimal timing of the postoperative RT. To answer this question, one randomized trial (RADICALS-RT) and one meta-analysis did not show that ART improved event-free survival when compared with early salvage radiation.^17^

Although the use of ADT concurrent with ART was selected by many panelists as a treatment for patients with high risk of recurrence after radical prostatectomy, this approach is still controversial. Until recently, there were no phase III trials favoring the use of concurrent ADT in the adjuvant setting.

## FOLLOW-UP: LOCALIZED AND LOCALLY ADVANCED, HIGH-RISK PROSTATE CANCER

In a patient with high-risk and/or locally advanced PCa, who underwent surgery with a curative intent, most of the panelists (60.76%) recommended following the majority of patients by anamnesis, physical examination including digital rectal examination, and PSA every 3-6 months for 5 years then every year. For the same patient, in an area of limited resources, the panel reached consensus (77.22%) in recommending follow-up by anamnesis, physical examination including digital rectal examination, and PSA every 3-6 months for 5 years then every year.

After RT (any form) with curative intent (with or without ADT) in a patient with high-risk and/or locally advanced PCa, the panel reached consensus (76.62%) in recommending following the majority of patients by anamnesis, physical examination including digital rectal examination, and PSA every 3-6 months for 5 years then every year. Consensus (82.05%) was also reached to follow up patients the same way in areas of limited resources (Data Supplement).

After definitive therapy with curative intent for high-risk PCa, most of the panel (64.02%) indicated they would never order imaging as follow-up for the majority of patients. For the same patient, in an area of limited resources, the panel reached consensus (85%) indicating that they would not order imaging at follow-up.

In conclusion, definitive treatment of the primary tumor for high-risk PCa is achieved either surgically or with RT and ADT. ADT alone is inadequate. Several factors such as life expectancy, tumor stage, lymph node involvement, and regional metastasis may affect the decision-making process between surgery and radiation. In scarce-resource settings, given the low availability of IMRT, 3DCRT, or image-guided radiation therapy combination of hormonal therapy and EBRT if conformal RT is available, particularly conventional conformal external beam, as the minimum accepted technique or radical prostatectomy in expert hands may be the best available option for the treatment and management of these patients. Nonetheless, even in areas of limited resources, when the only available RT is a cobalt machine, this treatment method is not recommended, and alternate surgical or medical options should be sought to manage the high-risk patient with disease confined or not confined to the prostate. Regarding long-term ADT, it was determined that orchiectomy may be an alternative for areas of resource limitations. Radical prostatectomy could translate into a more cost-effective strategy in areas of scarce resources, with limited access to RT. Open surgery is deemed to be adequate.
